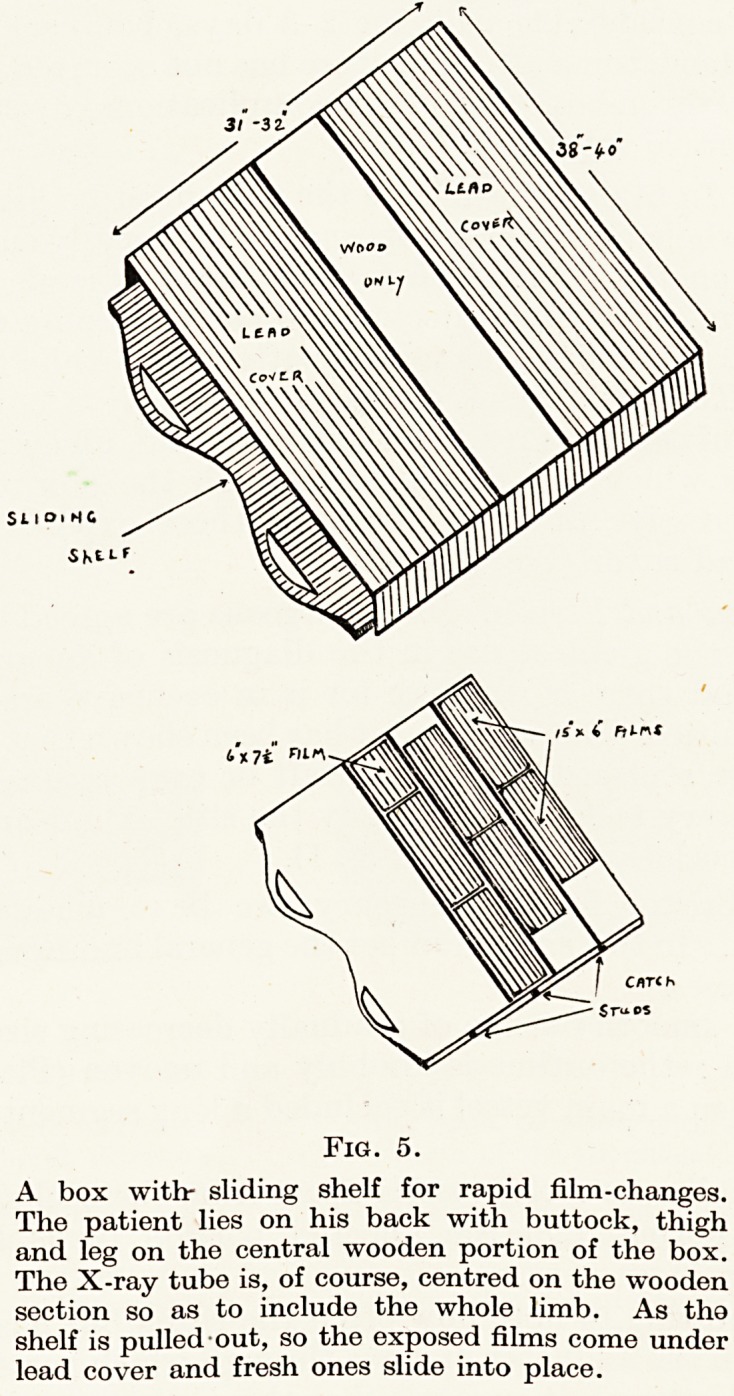# Arteriography of the Lower Limbs

**Published:** 1949-04

**Authors:** H. D. Moore

**Affiliations:** McKenzie MacKinnon Research Fellow, from the Department of Surgery, The University of Bristol


### ARTERIOGRAPHY OF THE LOWER LIMBS

BY

H. D. Moore, F.R.C.S.

*McKenzie MacKinnon Research Fellow, from the Department of Surgery, The University of Bristol.*

Arteriography consists in taking X-rays of arteries, the outlines of which are rendered visible by the injection of an opaque medium. The first attempt was made on animals in 1910, bismuth in oil being used. Since then :[1]

1923 Lipiodol used in dogs
Strontium bromide in humans

1924 50 per cent. sodium iodide

1927 Lipiodol in lower limbs of humans

1930 Lipiodol emulsion

1931 Thorotrast in the aorta and lower limbs ; organic iodides used also

1932 Thorotrast for cerebral vessels.

The media now in use are three. Thorotrast is favoured by many.[2, 3] It has the advantage of greater opacity than the iodides and is usually non-irritant : but Lambert[4] in 1935 had a case of gangrene of the leg ; and other disasters reported are death from syncope[5] and suppuration in the groin after extra-vascular injection.[6] The great danger, however, is that it is radioactive : ill results from this have been reported.[9]

Sodium iodide is likely to produce severe reactions and much pain, but it is still used for the aorta.[10, 11]

Organic iodides are the substances now usually employed. The most favoured is diodone (diodrast, Pyelosil). It is prepared in solutions of 35, 50 or 70 per cent. No disasters following its use have been published. Local reactions depend on the concentration : the 35 per cent. solution may produce a feeling of mild heat, the 70 per cent. of great heat, but this passes off quickly. The patient should be warned or he may move and spoil the X-ray. General reactions are very rare : syncope, vomiting, urticaria, etc., have been reported,[12] and it is advisable to have adrenaline at hand. An ocular " sensitivity " test consists of placing a drop of the solution in the conjunctival sac—if no redness occurs within 1½ minutes the test is " negative "[13]: the value of this is doubtful.[14] Disasters have been reported with other organic iodides, such as Tenebryl,[7] and uroselectan.[8]

*Method.* The medium may be injected either by exposing the vessel surgically or by percutaneous puncture. The former has the merit of direct control of the puncture site and of the vessel. The injection may be made either without disturbing the artery[15] or after placing around it a tape to control the amount of blood entering during and after the injection, and thereby the dilution of the medium. It is, however, an operation, and one which can be troublesome if small tributaries are torn : and therefore to be avoided if a simpler and safe procedure is available.

Percutaneous puncture has the theoretic disadvantages that the vessel may bleed from the puncture site and that the opaque medium may escape into the tissues. But in practice many hundreds of arterial punctures have been made without any trouble at all : if reasonable precautions are taken, nothing does escape from the vessel—and even if it does, no great harm appears to result when Pyelosil is used. During the injection, one may attempt to control the amount of blood entering the artery by proximal pressure[17] or one may rely on a rapid injection and speedy change of X-ray films to record the passage of the medium down the arteries of the limb.

Before making the injection the state of the vessels and the site of the upper end of the obstruction in the artery is determined clinically and by oscillometry. It is important to know this, for if most of the main vessel is fairly normal 70 per cent. solution is used (Plate V, Fig. 1) : but if the block is high in the main vessel or there is narrowing of the vessels (e.g. with atheroma), a 35 per cent. solution is sufficient, for not enough blood enters it to dilute the medium as it is injected (Plate VI, Fig. 4). Knowing the site allows one also to place the films so that the view of the obstruction does not fall at the junction between two of them.

The area over and near the artery is infiltrated with procaine solution. A small nick is made in the skin and the needle (1½ to 2 ins. long, 18 B.W.G.); is gently pushed in without the syringe, guided by one finger on the vessel above the puncture and one below. When the artery is reached, the needle transmits the impulse and, if it is allowed to rest on the vessel, it inserts itself with a little jerk, and the bright blood squirts out.

If the artery goes into spasm when pricked, which does not occur if sufficient procaine has been used, the blood does not squirt out very forcibly nor does it seem as bright as might be expected. This may make the injection difficult, and the visualization of the distal vessels poor. It is surprising with what force venous blood comes from the femoral vein if this is entered : but it is usually fairly

PLATE V

FIG. 1.

Arteriogram using 20 c. cm. of 70 per cent. Pyelosil.
G.H., male, 45 years, under the care of Professor Milnes Walker, suffering from Buerger's disease with intermittent claudication, but a good blood-supply to the skin. The film shows normal appearance of the vessels above and below the block, and the collateral circulation. In July, 1947, the affected segment of artery was excised, but without improvement. The veins seemed normal. In October, 1948, arteriograms using 20 c. cm. of 35 per cent. Pyelosil gave poor films, but showed the vessels to be unchanged. Lumbar sympathetic block with procaine produced good dilatation of the skin vessels but made the claudication worse. Sympathectomy was, therefore, inadvisable, but the missing segment of artery was replaced by a vein-graft.

FIG. 2.

Arteriogram using 20 c. cm. of 35 per cent. Pyelosil.
T.C., male, 60 years, under the care of Mr. Croot. Mild symptoms: the film shows the moth-eaten appearance due to moderate atheroma, and the rapid narrowing of the vessels of the leg. On the other side the superficial femoral artery was thrombosed, and the symptoms were much more severe. A 35 per cent. solution of Pyelosil was thought to be sufficient because of the clinical atheroma and the reduced readings with the oscillometer.

Fig. 3.

Arteriogram, using 20 c. cm. of 35 per cent. Pyelosil.
C.C., male, 76 years, with advanced atheroma of the femoral arteries; showing the knobbly, uneven appearance of the lumen of both the superficial and deep vessels. The main vessels of the leg were all occluded, and the supercfiial femoral narrowed, which accounts for the good picture with 35 per cent. Pyelosil and for the outline of the profunda femoris, which rarely shows when the superficial artery is patent.

Fig. 4.

Arteriogram using 20 c. cm. of 35 per cent. Pyelosil.
S.W., male, 60 years, with atheromatous occlusion of the superficial artery. This patient had gangrene of the foot and lower leg. The superficial femoral artery is entirely blocked and the blood is flowing via the profunda femoris and a very meagre collateral circulation. The popliteal artery is patent but obviously affected. Amputation below the knee was successful.

obvious that this has happened both because of the dark colour of the blood and because the needle has to be pushed in much further than expected.

After entering the artery, the needle is advanced a few millimetres and normal saline is injected to be sure all is well. 20 c. cm. of Pyelosil is then injected as rapidly as possible ; speed is of the utmost importance if the blood-flow is not controlled—hence the short, wide-bore needle. If it has been impossible to advance the needle within the vessel, local extravasation of Pyelosil may occur. This causes considerable pain for 2–3 days, but usually no other ill effects. Hæmatoma after puncture has not occurred in our cases : neither have thrombosis nor other complications, even in patients with considerable atheroma.

*X-rays.* In taking the X-rays the tube must be high enough to include the whole limb in one exposure : this usually entails placing the patient on the floor. The first exposure is made after about 15 to 16 c. cm. have been injected and the remaining two or three films are taken as quickly as possible after that, while the injection continues. To obtain quick changes, we employ a box which will hold one set of cassettes ready for exposure, and, under a lead cover, two or three which can be pulled through the box, thus rapidly exposing successive films and bringing those exposed once again under the lead cover (Fig. 5, p. 40).

*Indications and Results.* Most surgeons are agreed that arteriography is of the greatest use in the diagnosis of aneurysms. It is often said that there is no place for it in occlusive arterial disease of the extremities. But now that it has been shown that an occluded artery can be replaced by a vein graft or even recanalized,[16] it is clearly necessary to know accurately the site, extent and nature of the arterial occlusion (Plate V, Fig. 1).

At the Bristol Royal Infirmary, we have made twenty-five arteriograms. In this small number the general findings for the main vessel are these :

Normal : smooth outline of gradually decreasing size.

Atheroma : the outline is knobbly and uneven (Plate VI, Figs. 3, 4), and when a main vessel is occluded a long segment tends to be affected.

Buerger's disease : the vessels apart from the occluded segment seem to be normal and the affected portion tends to be short (Plate V, Fig. 1).

It is interesting to note how often the thrombus has one end at the opening in the adductor magnus through which passes the superficial femoral artery : it suggests that this is the site of origin, with occlusion spreading up or down as the case may be (Plate V, Fig. 1, Plate VI, Fig. 4).

This work was done with the help of a Beaverbrook Fellowship, which I gratefully acknowledge. I would like, also, to thank Professor Milnes Walker, M.S., F.R.C.S., and Mr. Croot, F.R.C.S., for allowing me to examine their cases and to publish this report and for their interest and encouragement in this work.

FIG. 5.

A box with sliding shelf for rapid film-changes. The patient lies on his back with buttock, thigh and leg on the central wooden portion of the box. The X-ray tube is, of course, centred on the wooden section so as to include the whole limb. As the shelf is pulled out, so the exposed films come under lead cover and fresh ones slide into place.

### REFERENCES

[1] Edwards, E. A.—*New Engl. Jour. Med.*, 1933, **209**, 1337.

[2] King, F. M.—*Radiography*, 1945, **11**, 77.

[3] List, C., Burge, C., and Hodges, F.—*Radiol.*, 1945, **45**, 1.

[4] Lambert, M. O.—*Bull. et Mem. Soc. Nat. de Chir.*, 1935, **61**, 173.

[5] Wertheimer and Frieh.—*Ibid.*, 361.

[6] Desplas, B., and Reboul, H.—*Ibid.*, 16.

[7] Leveuf, M. J.—*Ibid.*, 7.

[8] Leclerc, G.—*Ibid.*, 17.

[9] *Jour. Am. Med. Assoc.*—" Report of the Council on Pharmacy and Chemistry," 1932, **99**, 2183.

[10] Wagner, F. B.—*Jour. Urol.*, 1946, **56**, 625.

[11] Nelson, W. A.—*Surg. Gyn. Obstr.*, 1942, **74**, 655 ; 1945, **53**, 521.

[12] Pendergrass, E., Chamberlin, G., Godfrey, E., and Burdick, E.—*Amer. Jour. Roent.*, 1942, **48**, 741.

[13] Archer, V., and Harris, I.—*Ibid.*, 763.

[14] Robins, S.—*Ibid.*, 767.

[15] Learmonth, J. R.—*Lancet*, 1944, **2**, 745.

[16] Bazy, L., and Champy.—*Bull. Acad. Nat. Med. Baris*, 1948, **132**, 159.

[17] Allen, E. V., Barker, N., and Hines, E.—*Peripheral vascular diseases*, Saunders, 1947.

## Figures and Tables

**Fig. 1. f1:**
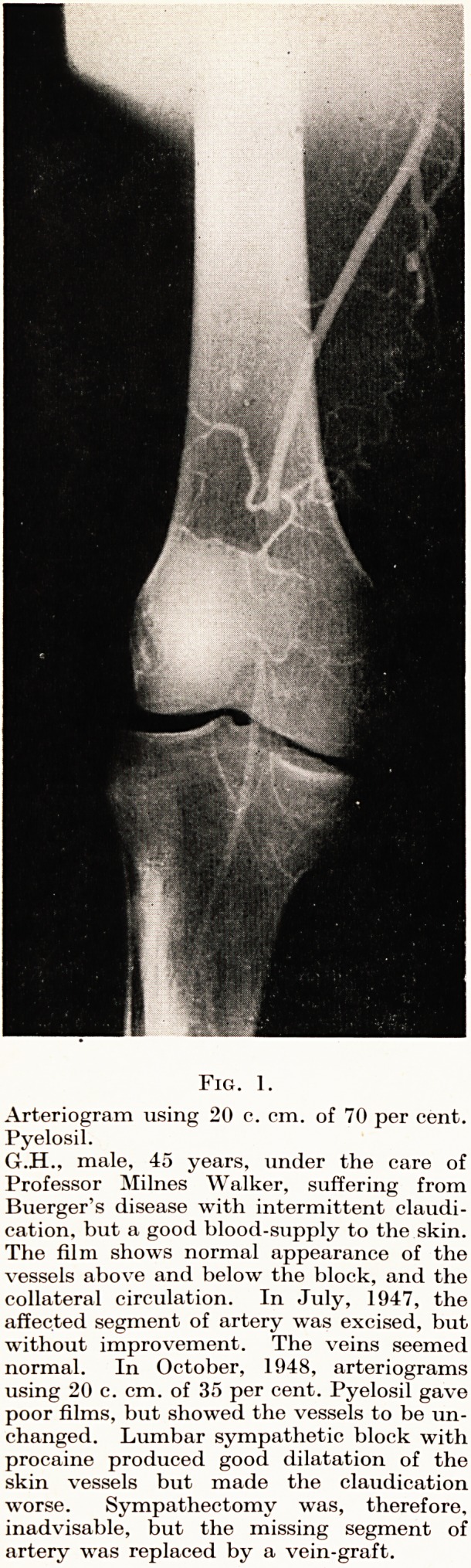


**Fig. 2. f2:**
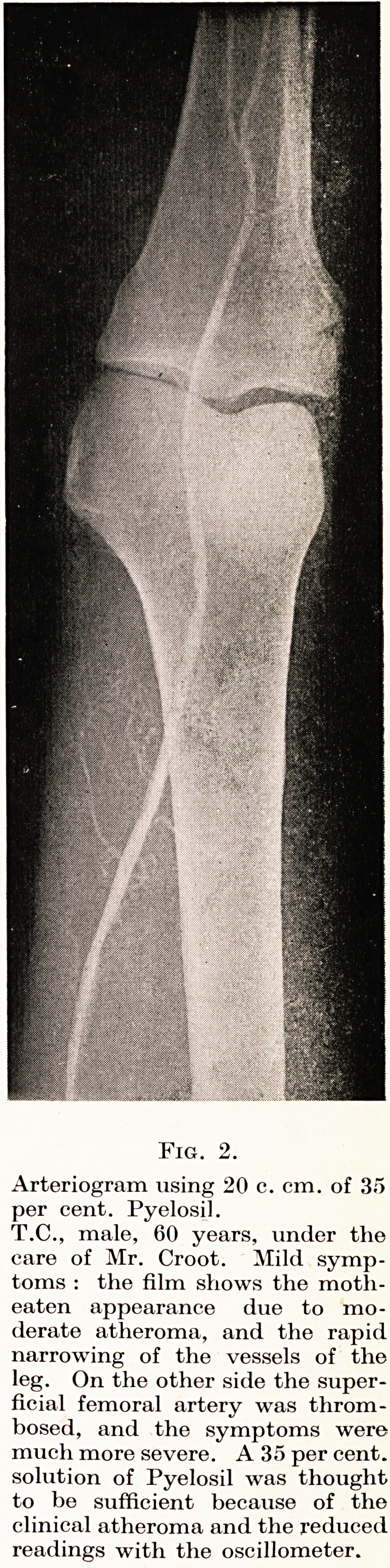


**Fig. 3. f3:**
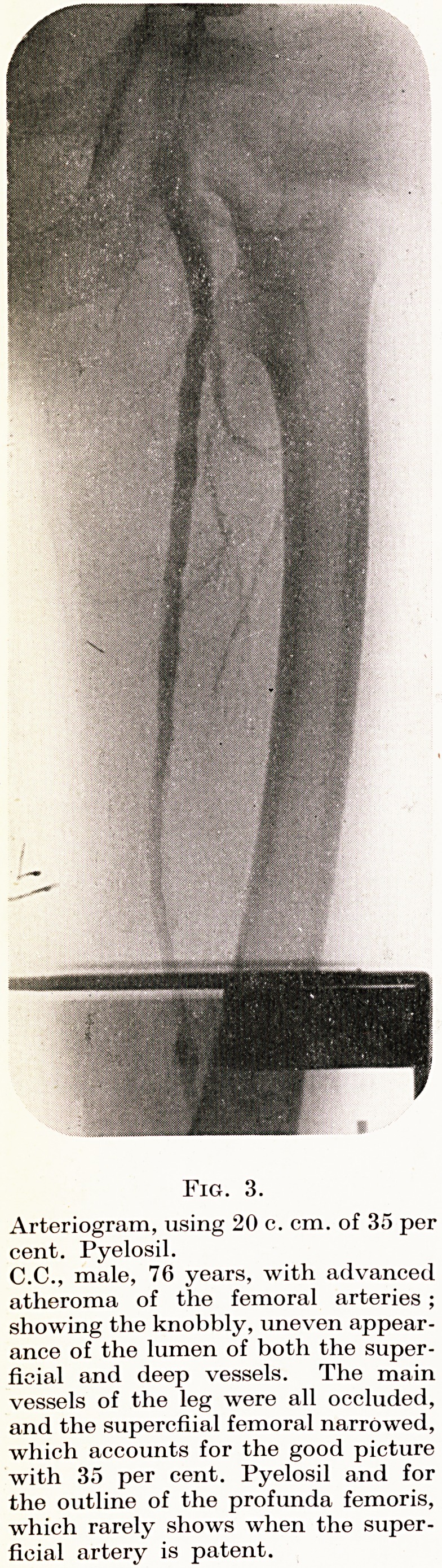


**Fig. 4. f4:**
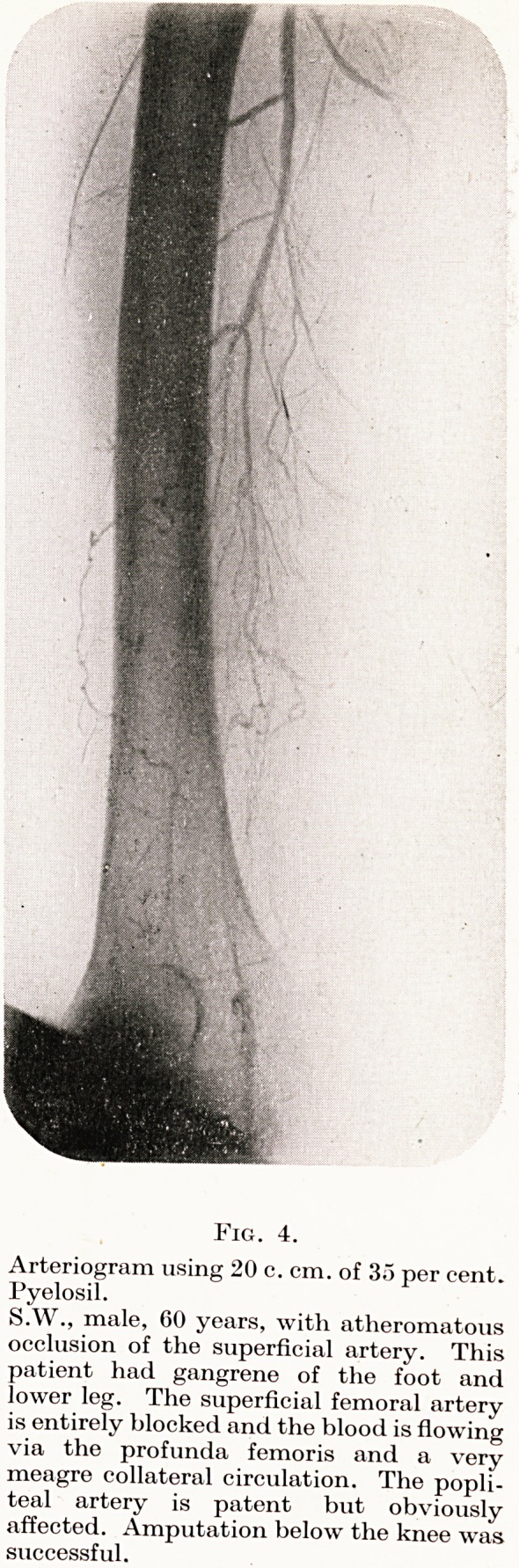


**Fig. 5. f5:**